# High-Throughput Phenotyping Approach for the Evaluation of Heat Stress in Korean Ginseng (*Panax ginseng* Meyer) Using a Hyperspectral Reflectance Image

**DOI:** 10.3390/s21165634

**Published:** 2021-08-21

**Authors:** Eunsoo Park, Yun-Soo Kim, Mohammad Kamran Omari, Hyun-Kwon Suh, Mohammad Akbar Faqeerzada, Moon S. Kim, Insuck Baek, Byoung-Kwan Cho

**Affiliations:** 1 Department of Biosystems Machinery Engineering, College of Agricultural and Life Science, Chungnam National University, Daejeon 34134, Korea; besoo12@cnu.ac.kr (E.P.); kamran.umari@o.cnu.ac.kr (M.K.O.); akbar.faqeerzada@o.cnu.ac.kr (M.A.F.); 2 R&D Headquarters, Korea Ginseng Corporation, Daejeon 34128, Korea; gintechkim@kgc.co.kr; 3 Department of Life Resources Industry, Dong-A University, Busan 49315, Korea; davidsuh79@dau.ac.kr; 4 Environmental Microbial and Food Safety Laboratory, Agricultural Research Service, United States Department of Agriculture, Beltsville MD 20705, USA; moon.kim@usda.gov (M.S.K.); insuck.baek@usda.gov (I.B.); 5 Department of Smart Agriculture System, Chungnam National University, Daejeon 34134, Korea

**Keywords:** near-infrared hyperspectral imaging, non-destructive measurement, spectral analysis, plant phenomics, ginseng, stress monitoring

## Abstract

*Panax ginseng* has been used as a traditional medicine to strengthen human health for centuries. Over the last decade, significant agronomical progress has been made in the development of elite ginseng cultivars, increasing their production and quality. However, as one of the significant environmental factors, heat stress remains a challenge and poses a significant threat to ginseng plants’ growth and sustainable production. This study was conducted to investigate the phenotype of ginseng leaves under heat stress using hyperspectral imaging (HSI). A visible/near-infrared (Vis/NIR) and short-wave infrared (SWIR) HSI system were used to acquire hyperspectral images for normal and heat stress-exposed plants, showing their susceptibility (Chunpoong) and resistibility (Sunmyoung and Sunil). The acquired hyperspectral images were analyzed using the partial least squares-discriminant analysis (PLS-DA) technique, combining the variable importance in projection and successive projection algorithm methods. The correlation of each group was verified using linear discriminant analysis. The developed models showed 12 bands over 79.2% accuracy in Vis/NIR and 18 bands with over 98.9% accuracy at SWIR in validation data. The constructed beta-coefficient allowed the observation of the key wavebands and peaks linked to the chlorophyll, nitrogen, fatty acid, sugar and protein content regions, which differentiated normal and stressed plants. This result shows that the HSI with the PLS-DA technique significantly differentiated between the heat-stressed susceptibility and resistibility of ginseng plants with high accuracy.

## 1. Introduction

Korean ginseng (*Panax ginseng* Meyer) is an herbal medicine plant and it is known to have high pharmacological efficacy in its roots. Representative pharmacological functions are known for their immune enhancement effects and fatigue recovery [1]. Unlike many other plants, the ginseng plant is sensitive to various environmental conditions, such as temperature, soil and moisture, but is particularly sensitive to temperature. The ginseng plant has a maximal photosynthetic rate at about 21–25 °C. However, photosynthetic activity decreases when the temperature rises above 25 °C and the quality and production decrease as the temperature rises above 30 °C [2].

The increase in Earth’s average temperature is becoming a threat to crop production, including ginseng production [3]. Continual exposure to high temperatures, especially during the summer, leads to physiological disorders resulting in yellowing and defoliation of ginseng plants. Yellowing and defoliation result in the deterioration of crop quality, making it difficult to recover its productivity [2].

Many researchers are developing high temperature-resistant ginseng varieties that can maintain quality even under high-temperature environments [4,5,6,7]. However, this development requires the selection and verification of the resistance of various ginseng varieties. Since the selection and verification procedures cost a lot of time and money, it is essential to have phenotypic high-throughput systems and data processing techniques to verify the resistance status of ginseng [8,9,10].

In general, plant phenotyping through RGB color images effectively quantifies the change of state observed with visible information. In large quantities, color image data can be obtained by observing the size, color and morphological characteristics of the target crop, which are useful for breeding or cultivation management of crops [11]. However, there is a limitation in analyzing the physicochemical properties of plants based on color images. When ginseng is exposed to heat stress, various physicochemical changes occur internally, which are very difficult to observe with RGB color images. By the time the stress symptoms are observed via color images, the ginseng has already accumulated considerable damage inside and out.

Among the high-throughput non-destructive technologies, hyperspectral imaging (HSI) indirectly infers the state of biochemical changes in crops to determine the stress level [11,12,13]. HSI can show a chemical or spectroscopic image containing spatial information by combining spectroscopy of a single point into a two-dimensional image [14,15]. Plant phenotyping requires the observation of a pattern of two or more dimensions and hyperspectral image information can be beneficial for extracting the phenotype [16]. Therefore, HSI is a useful technology to measure heat stress in ginseng, as well as to detect the resistance phenotype. A recent study showed that the detection of drought stress in corn was successfully measured using HSI in visible and near-infrared (Vis/NIR) imaging [17]. Hyperspectral fluorescence, Vis/NIR and short-wave infrared (SWIR) imaging showed the potential to detect a salt stress reaction and measure the biochemical reaction of crops such as soybeans, wheat barley, lettuce, etc. [18]. In addition, the moisture and nitrogen content of corn and soybean leaves can be predicted and the possibility of P, K, Mg, Ca, S, Fe, Mn, Cu and Zn prediction was reported using the wavelength range from 550 nm to 1700 nm [19,20]. A recent study showed that there is a correlation between the HSI of rice and the actual protein content and his, in a range from 350 nm to 2500 nm, could be used as a phenotypic tool for genome-wide association studies [21].

The purpose of this study is to identify visible and near-infrared wavelengths that can be indirectly observed as phenotypic factors related to resistibility and susceptibility of heat stress in ginseng plants at the laboratory level. The spectral model related to heat stress was developed by multivariate analysis using spectral data extracted from HSI and was used as a primary method of wavelength extraction. The extracted wavelengths were compared concerning the biochemical patterns related to heat stress in ginseng to determine the phenotypic correlation. In addition, the potential of high-throughput screening of heat stress resistance ginseng using hyperspectral imaging technology was investigated.

## 2. Materials and Methods

### 2.1. Plant Materials, Environmental Conditions and Heat Treatment

One-year-old seedlings (harvested in mid-March 2020) of three different P. ginseng varieties used in the experiment were provided by the R&D Headquarters of the Korea Ginseng Corporation in Daejeon. One of the three varieties (Chunpoong) is susceptible to high temperature and the other two (Sunil and Sunmyoung) are resistant varieties. For this study, each seedling was transplanted into a small pot and grown in a growth chamber (HB-301S-3, Hanbaek Scientific Co., Bucheon, Korea), with 22 ± 1 °C, 60–70% humidity, 16:8 photoperiod, with 15,000 Lx light intensity for four weeks.

**Figure 1:**
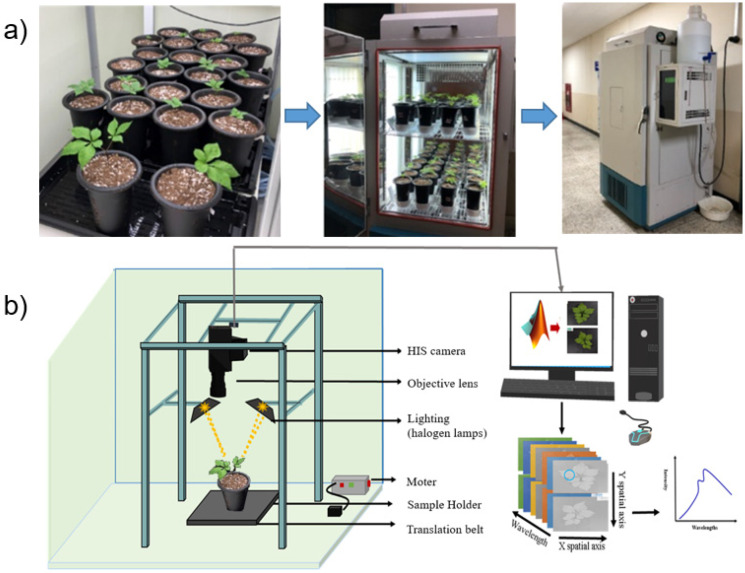
Heat stress treatment of ginseng plants in the growth chamber (**a**) and HSI system components (**b**).

Before heat treatment, the plants were measured using an HSI system as a control. The ginseng samples were selected to have relatively similar growth conditions, with a total of 52 samples, including Chunpoong (22 samples), Sunmyoung (12 samples) and Sunil (18 samples) varieties. The plants were then exposed to a temperature of 30 ± 1 °C in the growth chamber for four weeks. After heat treatment, hyperspectral data and color images were collected and used for further analysis. The ginseng growth conditions and heat treatment in the growth chamber are shown in Figure 1a.

### 2.2. HSI Systems and Image Acquisition

In this work, two laboratory-based line scan HSI systems in different wave ranges, Vis/NIR (400–1000 nm) [22] and SWIR (1000–1800 nm) [23], were used in reflectance mode to collect the hyperspectral images of plant samples (Figure 1b). The spectral intensity beyond 1800 nm was significantly low and noisy, compared to the range of shorter wavelength. So, the spectral range beyond 1800 nm was not included for further analysis. When the operating system was stable, ginseng plants were manually placed on the translation stage and scanned line-by-line with the HSI camera. The HSI cameras were installed on standing platforms for both operating systems and began to collect HSI data as the ginseng plants approached the field of view of the camera. The distance between the camera and the samples was set to 26 cm. The ginseng plants were scanned in Vis/NIR and SWIR at a scanning speed of 6 mm/s. The collected raw HSI data were stored in a three-dimensional format comprising two spatial and one spectral dimension. After acquiring the HSI of plant samples, white and dark reference images were acquired from both VIS-NIR and SWIR for image calibration.

### 2.3. Data Extraction

**Figure 2:**
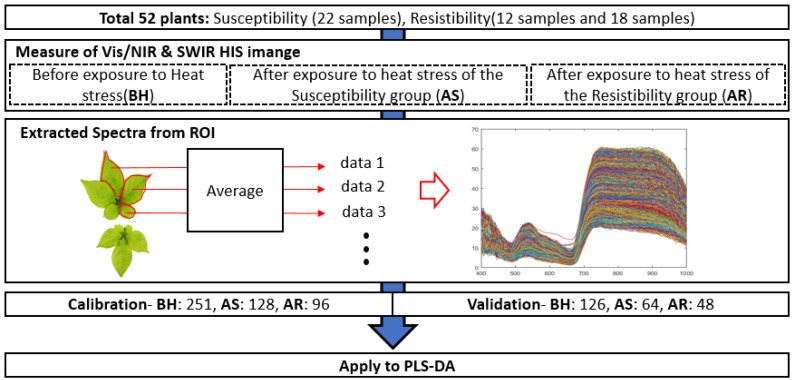
Diagram of data extraction process.

Vis/NIR and SWIR images of 52 samples were obtained (Figure 2). The obtained spectrum was extracted from the leaves of the ginseng plant in the image and the average of each leaf spectrum was determined as one sample data (about 20,000-48,000 pixels). The spectral data before heat stress exposure were a total of 377 samples (161 Chunpoong, 89 Sunmyoung and 127 Sunil). In addition, the spectral data after heat stress exposure included a total of 337 samples (144 Chunpoong, 87 Sunmyoung and 106 Sunil). Based on the obtained spectral data, ginseng plants were divided into three groups—all plant samples before exposure to heat stress (BH), the susceptibility group after exposure to heat stress (AS) and the resistibility group after exposure to heat stress (AR). For calibration, the total number of samples was divided into 251 samples of BH, 128 samples of AS and 96 samples of AR. For validation, the total number of samples was divided into 126 samples of BH, 64 samples of AR and 48 samples of AS. Matlab (Version R2019a, Mathworks, Natick, MA, USA) was used for all data processing.

### 2.4. Multivariate Analysis for Classification

#### 2.4.1. Linear Discriminant Analysis (LDA)

This study observed the spectral differences before and after exposure to high temperatures by three groups (BH, AS and AR) based on LDA. LDA was used to determine the optimal linear correlation of the three groups. LDA is a supervised classifier that finds a linear correlation between two or more groups. The correlation among the three groups was verified by the best projection value (BPV) of LDA.

#### 2.4.2. Partial Least Squares Discriminant Analysis (PLS-DA)

Based on the optimal correlation represented by LDA, PLS-DA was used to develop a classification model for the control and stress of ginseng plant groups. PLS-DA is a discriminant analysis method that is a modified version of partial least squares regression (PLSR) and is mainly used for classification purposes. PLSR is a regression method that finds the model with the most significant correlation among latent variables (LVs) of input (hyperspectral data) and output data (references values for ginseng plant based on heat stress states) [24,25]. Since LDA is known to produce very similar results to PLS-DA [25,26], the BPV of LDA was applied as three groups of dependent variables (reference values) of PLS-DA.

#### 2.4.3. Main Wavebands Selection for the Classification

Waveband selection is an essential step in spectral data analysis that can be used to reduce the high dimensionality of hyperspectral data. In general, the selection of informative variables can often provide a better and simpler prediction of the wavebands containing the most critical information. In addition, the elimination of unnecessary, irrelevant and noisy wavebands can improve model accuracy and decrease model complexity. To obtain the main wavebands (phenotype) related to high-temperature exposure, the variable importance in projection (VIP) and successive projection algorithm (SPA) methods were applied to extract the main wavebands. An ensemble filtering algorithm that combined the VIP and SPA methods was applied. VIP represents the contribution of each waveband determined by the optimal LVs of PLS-DA as a VIP score. The wavebands with a greater than or equal to VIP score were determined to be the main wavebands [27]. The equation for calculating the VIP score of a variable j is as follows:

(1) $${\mathrm{VIP}}_{j}=\sqrt{\frac{\sum_{F=1}^{F}W_{jf}^{2}\;.\;SSY_{f}\times J}{\;SSY_{total}\times F}}$$

where *W_if_* is the weight value for component *f* of variable *j*; *SSY_f_* is the sum of the squares of explained variance for the *f*th component, *J* is the number of variables, *SSY_total_* is the total sum of the squares for the dependent variable and *F* is the total number of components.

The SPA obtained, in the PLS-DA-based model, the main wavebands based on the VIP score. SPA is an algorithm that prevents collinearity and selects the minimum overlapping wavebands in a multiple linear regression model such as PLS-DA. This process can help reduce data noise and extract the main wavebands [28,29]. In this study, VIP was applied to select main wavelength regions with high weight in the PLS-DA model of full wavelengths; then, SPA was applied to extract less than 20 major wavelengths in the VIP result.

## 3. Results

### 3.1. Spectral Profile of Ginseng

**Figure 3:**
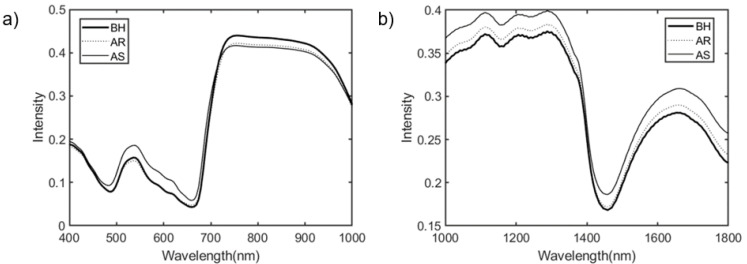
Averaged spectra of Vis/NIR (**a**) and SWIR (**b**) of control and stressed ginseng plants. BH, before exposed to heat stress; AR, after exposed to heat stress group about resistibility; AS, after exposed to heat stress group about susceptibility.

The average spectra of Vis/NIR and SWIR of the BH, AR and AS groups of ginseng plants are shown in Figure 3. In the visible region (from 400 nm to 700 nm), BH and AR showed very similar average spectral intensities (Figure 3a). On the other hand, in the case of AS, it was observed that the intensity increased more than BH. This observation indicated that BH and AS could be distinguished when exposed to heat stress in the visible area. In the NIR region (700 nm–1000 nm), an intensity difference between the BH and the heat stress-exposed groups (AS and AR) was observed. Therefore, the NIR region can distinguish between samples before and after exposure to heat stress. In the SWIR region, the intensity of BH was lowest at 1000 nm–1300 nm and 1600 nm–1800 nm (Figure 3b). On the other hand, AR had a larger intensity than BH and AS had a larger intensity than AR. These results indicate a possibility of distinguishing between ginseng exposure and resistance to heat stress in the SWIR region.

The average spectrum can be observed as the overall spectrum pattern for each group by verifying the approximate spectral difference between groups through the differences in the spectral intensity and shape. However, if the graph of each group of the spectrum is shifted to the same form, it is possible that high deviation of the average of spectral data. Therefore, it is practical to compare differences in the shape of the spectrum. Figure 3a showed intensity and morphological differences in the visible region (450 nm–750 nm). Figure 3b generally shows differences in intensity by group, but it is difficult to identify these due to shifted tendencies. Therefore, the differences between groups were verified through PLS-DA.

### 3.2. LDA Analysis

**Figure 4:**
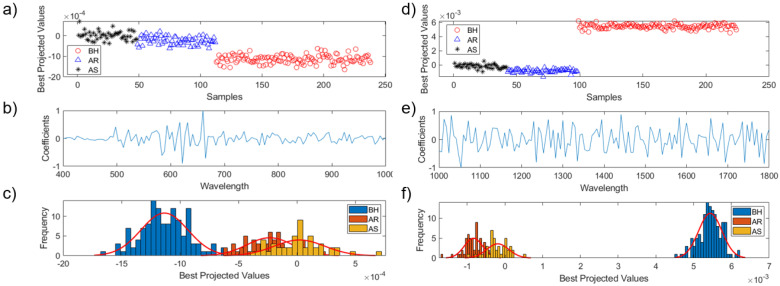
Averaged spectra of LDA results of Vis/NIR (**a**–**c**) and SWIR (**d**–**f**) of control and stressed ginseng plants. BH, before exposed to heat stress; AR, after exposed to heat stress group about resistibility; AS, after exposed to heat stress group about susceptibility.

Since, in this study, the dependent variables (Y reference values) were unknown, those could be used in the PLS-DA analysis to classify ginseng plant data into three different categories. In order to obtain correlations of three or more groups using PLS-DA, it is necessary to determine the numerical correlations that represent each group. This correlation is the same as the dependent variable (Y), when performing the supervised analysis of PLSR or PLS-DA [30]. However, there is a limitation in that the dependent variables cannot be identified quantitatively before and after the exposure to high temperature of susceptible and resistant plants. Therefore, Y values linearly extracted by best projection value (BPV) of LDA could distinguish the three groups. Figure 4 shows LDA analysis for Vis/NIR data and the LDA analysis results for SWIR. Figure 4a shows the optimal linear projections of the three groups found through the BPV of LDA and Figure 4b shows the relevant waveband coefficients of the LDA model. Figure 4c is a histogram based on the Y-axis value (BPV) of Figure 4a. When the histograms of each group were verified, the distribution of group values before (before heat stress, BH) and after heat stress (after resistibility, AR; and after susceptibility, AS) was clearly distinguishable. The AR and AS groups were found to be difficult to be distinguished from the condition of heat stress exposure. The representative value of each group was judged based on the central value of the normal distribution of the three histograms. If BH, an indicator of a normal plant group, is based on 0 and the most distant AS group is represented by 100, then the AR group can be represented by a numerical value of 74. Thus, the designated Y values for performing PLS-DA were 0, 74 and 100 for BH, AR and AS, respectively.

Figure 4d shows the distribution for each group and Figure 4e the coefficients of the waveband. Figure 4f shows the histogram of the distribution for each group when LDA was performed for SWIR data. Each group can be represented as 0 (BH), 89 (AS) and 100 (AR) based on the central value of the normal distribution of BF in Figure 4c. Therefore, these reference values were given to the respective SWIR spectral data for ginseng plants.

### 3.3. PLS-DA Model Results and Main Wavebands Coefficient

#### 3.3.1. Vis/NIR and SWIR Data Analysis

As a result of performing PLS-DA by applying BPV, the validation accuracy was 74.1%, as shown in Table 1, for the full wavebands (Full). On the other hand, when the Y value was applied as a sequential placement at regular intervals (SPRI; BF was 0, AR was 50, AS was 100), instead of BPV, it was possible to obtain improved results (76.7%), compared to BPV, as shown in Table 1. According to these results, when applying the unknown Y value of the PLS-DA model, it was expected that it would be advantageous to improve the accuracy by applying the value of the qualitative relationship, rather than the specific value of the BPV. When the full spectrum of BPV was explored by the ensemble VIP and SPA, 128 bands could be reduced to 18 bands. The accuracy of validation was 74.9%, which was not different from the full spectrum. Therefore, it can be seen that VIP and SPA ensembles are viable methods to extract the main wavebands.

On the other hand, the full spectrum of SPRI was explored by ensemble VIP and SPA; it was reduced to 12 bands, 6 bands less than the main spectrum of BPV. The validation accuracy of the main spectrum of SPRI was 76.7%, which was 2.6% higher than the main spectrum of BPV. It can be seen that these results are affected by the order rather than the representative value of each data group when performing PLS-DA on three or more groups. Therefore, when applying the dependent variable (Y) of three or more unknown groups to apply PLS-DA, it was shown that the sequential correlation with the comparison group (BH) was the most important one.

**Figure 5:**
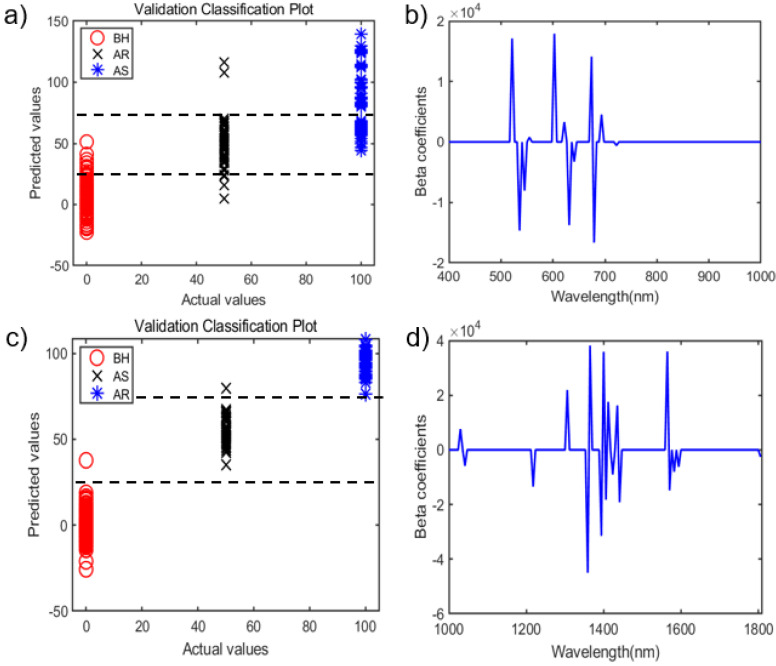
SPRI value of PLS-DA results of the 12 bands of spectra in Vis/NIR and of 18 bands of spectra in SWIR. (**a**) Classification plot of validation in Vis/NIR. (**b**) Beta coefficients of PLS-DA in Vis/NIR. (**c**) Classification plot of validation in SWIR. (**d**) Beta coefficients of PLS-DA in SWIR.

Figure 5a shows the results of the main wavebands of SPRI in Table 1. As shown in the Figure, in the Vis/NIR region, it is possible to distinguish between samples before and after heat stress. However, it is challenging to distinguish resistance to heat stress, because AR and BH overlapped. Figure 5b presents the results of the beta coefficients of the main wavebands. The main wavebands of the 12 bands were found to have a high weight of between 521 nm and 722 nm; the main wavebands of the 12 bands were 521, 535, 545, 555, 603, 622, 631, 641, 674, 679, 693 and 722 nm.

When performing PLS-DA by applying BPV to SWIR data, the validation accuracy was 93.8%, as shown in Table 1, for the full wavebands (Full). On the other hand, when the Y value was applied as SPRI (BF was 0, AS was 50, AR was 100) instead of BPV, it was possible to obtain improved results (100.0%), compared to BPV, as shown in Table 1. When the full spectrum of BPV was explored by ensemble VIP and SPA, 128 bands could be reduced to 18 bands. Furthermore, the accuracy of validation was 87.5%, which was 6.3% worse than the full spectrum. On the other hand, the full spectrum of SPRI was explored by ensemble VIP and SPA; it was reduced to 18 bands, as with the main spectrum of BPV. The validation accuracy of the main spectrum of SPRI was 98.9%—an improvement of 11.4%, compared to the main spectrum of BPV. It can be seen that these results are affected by the order rather than the representative value of each data group when performing PLS-DA on three or more groups. Therefore, when applying the dependent variable (Y) of three or more unknown groups to utilize PLS-DA, it was shown that the sequential correlation with the comparison group (BH) was the most important one.

As shown in Figure 5c, in the SWIR region, it is possible to distinguish between samples before and after heat stress regarding susceptibility and resistibility (Table 1, SWIR). The main wavebands of the 18 bands were 1030, 1042, 1218, 1306, 1359, 1365, 1394, 1400, 1406, 1412, 1424, 1435, 1441, 1565, 1571, 1582, 1594 and 1806 nm (Figure 5d).

#### 3.3.2. PLS-DA-Based Images for Heat Stress Detection

**Figure 6:**
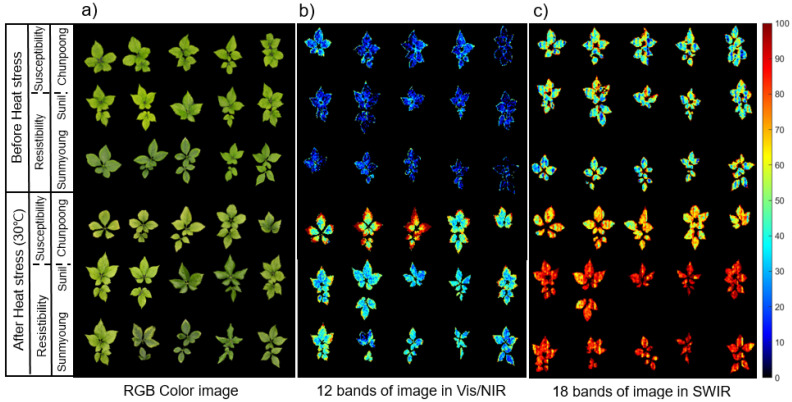
Comparison of color image and the resultant PLS-DA image using selected waveband images in Vis/NIR and SWIR. (**a**) Color image. (**b**) PLS-DA image constructed by 12 bands of spectral image in Vis/NIR and (**c**) 18 bands of spectral image in SWIR.

The resultant PLS-DA images (Figure 6) can be constructed from the linear combination of selected wavebands as indicated in Figure 5. The color bar of the Figure 6b,c indicates the resultant SPRI value of PLSD-DA for the pixel in the image. Figure 6b shows the imaging results based on the selected 12 wavebands of weights in Vis/NIR. A 12-waveband image can distinguish heat stress better than a color photo image (Figure 6a) by the edge of the leaf pattern (BF and AF). A 12-waveband multispectral image can distinguish samples before and after heat stress better than a color image and, in the multispectral image, the change of the edge of the leaf of AS was observed after heat stress. These phenomena are thought to show that AS varieties react sensitively to high temperatures, resulting in a decrease in chlorophyll from the edge of the leaves.

Figure 6c shows the results of imaging based on the selected 18 waveband weights in SWIR. An 18-waveband image can distinguish samples before and after heat stress in terms of susceptibility and resistibility, compared to an RGB color image. Therefore, the SWIR area is essential for distinguishing between heat stress exposure and resistance.

## 4. Discussion

In order to determine ginseng plants exposed to heat stress, hyperspectral Vis/NIR and SWIR image data that could represent the physicochemical information of ginseng plants were acquired and applied to LDA and PLS-DA for our prediction model. The applied results showed that SPRI (Vis/NIR, 76.7%; SWIR, 100.0%) obtained a higher validation accuracy than BPV (Vis/NIR, 74.1%; SWIR, 93.8%). This means that when three or more groups are applied to the PLS-DA analysis to create a classification model, the correlation has more useful information than each extracted Y value by LDA. This method can be used as an alternative to the method used when encoding the data of three classes of independent variables [31], such as “(1,0,0), (0,1,0), (0,0,1)”.

The main chemical components of ginseng plants can be altered, while the encountered heat stresses are 521 nm–722 nm in visible spectra. According to heat stress, the reaction of ginseng leaves is a change in chlorophyll content [4,6]. Among the selected main wavebands, the wavebands most closely related to chlorophyll are known to be at 545, 555, 674, 679 and 722 nm [32,33,34]. Chlorophyll is also closely related to nitrogen content. The wavebands of 545, 555, 641 and 722 nm are similar to the waveband range in the existing literature for nitrogen content [33,35].

A recent study showed that sugar metabolism and fatty acid accumulation are related to the heat stress sensitivity of ginseng [4]. The altered main chemical components of heat stress in the NIR area of ginseng plants were at 1030 nm–1806 nm. In the selected main wavebands, the wavebands associated with sugar were 1441 (O-H, sucrose) and 1582 nm (O-H, starch, or glucose) [36]. Most fatty acids have a -CH3 structure and the related wavebands are 1218, 1359 and 1394 nm [36]. It is also known that the metabolism of various proteins in ginseng plants is changed when exposed to high-temperature stress [36]. Protein-related wavebands are 1030 (-NH3) and 1571 nm (-CONH-) [36]. It is understood that the results of these experiments and literature review can be used as a phenotypic index that can sufficiently distinguish heat stress exposure and resistance. If a correlation analysis is performed between the selected waveband and actual components, it is expected that a heat stress model with spectroscopy can be developed more precisely. In addition, it is known that, under heat stress, the root of ginseng was disturbed with the growth along with the leaves [2]. Hence, linking the phenotypic indices of spectral information with ginseng leaves is expected to be of great support in indirectly predicting the developmental status of ginseng roots.

The PLS-DA image (Figure 6) in which the main wavelength is selected has the advantage of showing the chemical state of ginseng in a two-dimensional image. The chemical state of the ginseng plant represented by the image can more specifically show the progression of heat stress. In addition, PLS-DA images show potential for detecting heat stress conditions in large amounts of ginseng plants.

By comparing the performance of the Vis/NIR and SWIR regions, it is shown that the SWIR region is effective for distinguishing the susceptibility and resistibility of ginseng before and after exposure to high-temperature stress and when exposed to high-temperature stress. In addition, Vis/NIR has the advantage of being able to distinguish between samples before and after exposure to heat stress and with a small number of main wavelengths. Therefore, this technique is likely to be used for heat-stress monitoring in the field.

## 5. Conclusions

In order to develop a model for determining the susceptibility and resistibility to heat stress of ginseng, hyperspectral images of Vis/NIR and SWIR regions (400 nm–1800 nm) were measured. PLS-DA, VIP and SPA analyses were performed to select the main spectral wavebands. The Vis/NIR region showed 79.2% accuracy with 12 main wavelengths and the SWIR region showed 98.9% accuracy with 18 main wavelengths. The main wavelength of the Vis/NIR region was mostly related to the chlorophyll and nitrogen components and the SWIR region was related to the O-H (sucrose, starch, or glucose), -CH_3_, NH_3_ and -CONH- structures. These results indicate that heat stress affects photosynthesis and sugar metabolism and causes changes in the internal proteins to ginseng plants. Besides, the developed main spectral image performed better than a color image in distinguishing heat-stressed ginseng plants. These results indicate the potential of our method for use in heat stress-related growth management or resistant breeding.

## Figures and Tables

**Table 1:** Full spectrum of PLS-DA and main waveband extraction results of VIP–SPA ensemble analysis in the Vis/NIR and SWIR area.

				Total			Correct			Accuracy (%)			
				BH	AR	AS	BH	AR	AS	BH	AR	AS	Overall
Vis/ NIR	BPV	Full	Cal *	251	128	96	231	113	59	92.0	88.3	61.5	80.6
			Val *	126	64	48	110	57	22	87.3	89.1	45.8	74.1
		Main	Cal	251	128	96	229	113	57	91.2	88.3	59.4	79.6
			Val	126	64	48	108	57	24	85.7	89.1	50.0	74.9
	SPRI	Full	Cal	251	128	96	218	118	68	86.9	92.2	70.8	83.3
			Val	126	64	48	105	58	27	83.3	90.6	56.3	76.7
		Main	Cal	251	128	96	230	115	60	91.6	89.8	62.5	81.3
			Val	126	64	48	117	58	26	92.9	90.6	54.2	79.2
SWIR	BPV	Full	Cal	251	85	113	251	81	97	100.0	95.3	85.8	93.7
			Val	126	42	56	126	38	51	100.0	90.5	91.1	93.8
		Main	Cal	251	85	113	251	75	84	100.0	88.2	74.3	87.5
			Val	126	42	56	126	33	47	100.0	78.6	83.9	87.5
	SPRI	Full	Cal	251	85	113	250	85	113	99.6	100.0	100.0	99.9
			Val	126	42	56	126	42	56	100.0	100.0	100.0	100.0
		Main	Cal	251	85	113	250	85	112	99.6	100.0	99.1	99.6
			Val	126	42	56	125	41	56	99.2	97.6	100.0	98.9

* Sample number of calibration and validation, respectively.
